# Supplementary material for: Continuous generation of topological defects in a passively driven nematic liquid crystal
Source: Nat Commun. 2022 Nov 11;13:6855. doi: 10.1038/s41467-022-34384-5 (PMC9652398; doi:10.1038/s41467-022-34384-5)
Supplement: Supplementary file 1 — Supplementary Information [file 41467_2022_34384_MOESM1_ESM.pdf]

SUPPLEMENTARY INFORMATION
Continuous generation of topological defects in a passively driven nematic liquid crystal

Maruša Mur[1], Žiga Kos[2,1], Miha Ravnik[2,1], and Igor Muševič[1,2*]

[1] *Condensed Matter Physics Department, J. Stefan Institute, Jamova 39, 1000 Ljubljana, Slovenia.*
[2] *Faculty of Mathematics and Physics, University of Ljubljana, Jadranska 19, 1000 Ljubljana, Slovenia.*

*E-mail: igor.musevic@ijs.si

SUPPLEMENTARY NOTE 1: Transformation of a submerged liquid crystal drop to a freely floating film

A small drop of 8CB is deposited onto a glass slide with a needle, as shown in Supplementary Fig. 1(a). The liquid crystal (LC) drop is then covered with a large drop of 0.4 wt% water solution of 2-hydroxy-2-methylpropiophenone photoinitiator (HMPP). Because 8CB is a hydrophobic material, the drop of 8CB LC retracts to minimize its interface with water, as seen in Supplementary Fig. 1(b).

After the LC drop had been covered by a larger drop of water-HMPP solution for a couple of minutes, the thickness of water layer above the LC drop decreases because of evaporation. At one moment water film ruptures and the submerged LC drop is exposed to the ambient air (Supplementary Fig. 1(c)). The LC drop suddenly and unexpectedly 'explodes', pushes the water away and dramatically increases in diameter. Instead of a drop submerged in a water-HMPP solution, the LC transforms into a thin film, floating on top of the water-HMPP layer, as shown schematically in the right inset to Supplementary Fig. 1(d). In the left inset, the drop is shown under crossed polarizers, where the thin nematic film is more apparent. If the LC drop, initially covered with the water-HMPP solution, is covered with a glass plate, no such behavior is ever observed. In that case the HMPP slowly diffuses into the LC drop, gradually reducing its LC order until the drop eventually turns completely isotropic.

We used confocal microscopy to clarify the experimental geometry and obtain vertical cross-sections of the LC drop before and after the 'explosion'. Supplementary Fig. 1(e) shows confocal microscope image of the vertical cross-section of the LC drop (red) covered with the water-HMPP solution (cyan). We can clearly see that before the 'explosion' (Supplementary Fig. 1(e)) the LC drop had a convex shape (dark drop). It is also seen that after the 'explosion' the LC comes into contact with the outside air (not clearly visible, indicated with a dashed line) and the LC-water interface acquires a concave shape, as illustrated in the confocal cross-section in Supplementary Fig. 1(f). The core of the LC drop remains fixed to the bottom glass plate, but from there the LC spreads along the water surface and forms a thin film on top of the water layer. This is due to the different densities and surface tensions of the water and

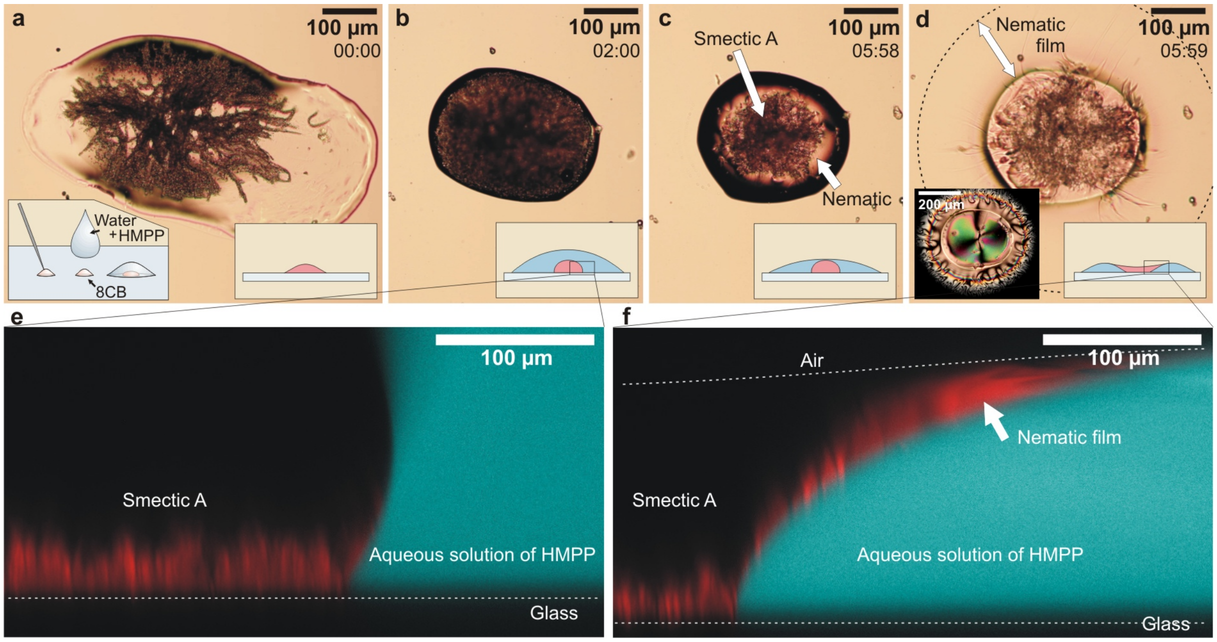

Supplementary Figure 1: Transformation of a LC drop submerged under a larger drop of a water solution of 2-hydroxy-2-methylpropiophenone photoinitiator (HMPP). (a) A drop of smectic-A LC 8CB is deposited on a glass plate. The first inset shows the sample preparation, the second one a schematic vertical cross-section of the sample. (b) After being submerged, the drop retracts to minimize its surface, as suggested by the schematic in the inset. (c) As the HMPP diffuses inside, the LC at the droplet edge changes its phase to nematic. The black edge is an optical artifact due to lensing of the drop. (d) Suddenly the drop 'explodes', see Supplementary Movie 1. A wide band of the turbulent nematic appears around the drop, while the smectic core remains motionless. The left inset shows a cross-polarized image of the drop, where the thin film is easily seen (see also Supplementary Movie 2). (e) Vertical cross-section of a smectic-A drop before the 'explosion' taken with a confocal microscope (aqueous solution of HMPP is dyed with FITC, the LC is dyed with Nile red). (f) Vertical cross-section of the drop after the 'explosion'. The nematic film can be seen spreading along the surface of the aqueous environment. The time in panels (a-d) is given the format *min:sec.*

the LC. In the confocal image, the nematic film on top of the water-HMPP solution appears brighter, as less of the light is scattered by the smoother nematic texture.

The HMPP dissolves well in 8CB, however, in water it is only soluble to a certain extent (1 wt%). Therefore, when the water solution of HMPP comes into contact with the LC, the HMPP starts to diffuse from the water into the LC. There it decreases the orientational order of the 8CB, and that causes a phase transformation of some parts of the smectic-A drop into the nematic phase at the same temperature. This is seen in Supplementary Fig. 1(c) as a dark rim of the nematic phase of 8CB, which is gradually forming at the edge of the smectic 8CB drop over a time span of several minutes, and the nematic-smectic-A interface is advancing towards the center of the smectic 8CB drop. When 2 wt% of HMPP is added to pure 8CB, the smectic-A to the nematic phase transition drops from 33°C to 25°C. If 5 wt% of HMPP is added, the 8CB becomes isotropic already at 25°C. This indicates that the incorporation of HMPP molecules causes a great deal of disruption to the local orientational ordering of the 8CB molecules.

SUPPLEMENTARY NOTE 2:
Anisotropy of + and − defect dynamics during annihilation

Supplementary Figure 2 shows velocity distributions of +1 and −1 defects during the annihilation process. Here, the velocity is measured in the coordinate system that is at rest (i.e. the camera). One can see that the negative defects move apparently faster that the positive ones during annihilation, which is not in agreement with many experimental reports, where positive defects always moved faster than the negative ones. This can be explained by the overall drift of both positive and negative defects due to the background flow of the LC, which actually carries both defects. It is also interesting to note that the positive +1 defects are always ahead the −1 defects and they slow down during the annihilation, as they are pulled back by the negative neighbour, which speeds-up for the same reason. This could be the reason for the apparent higher measured velocity of negative defects in laboratory reference frame during annihilation.

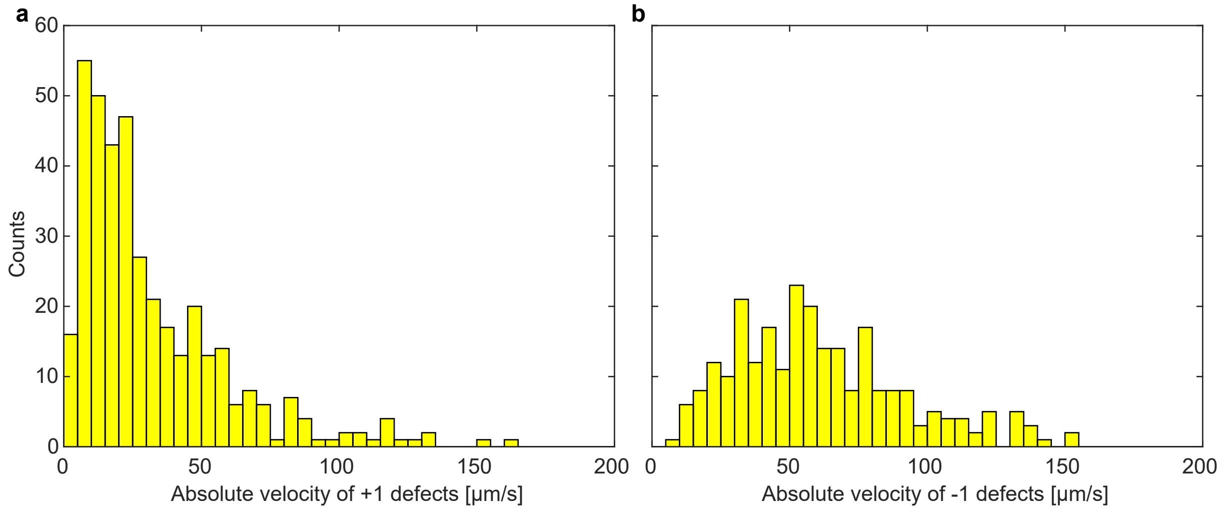

Supplementary Figure 2: Anisotropy of + and − defect dynamics during annihilation. (a) Distribution of absolute velocities of +1 defects during annihilation of a pair of oppositely charged defects. (b) Distribution of absolute velocities of −1 defects during annihilation.

SUPPLEMENTARY NOTE 3: Numerical simulations

Model equations

We describe the deformation of the nematic alignment field in the vortex flow and in the irregular flow by solving the dynamic equations for the flow field, concentration field of the HMPP, and the nematic alignment field.

Navier Stokes equations

Fluid velocity obeys the Navier Stokes equations for incompressible flow

$$\rho \left(\frac{\partial}{\partial t} + \mathbf{v} \cdot \nabla \right) \mathbf{v} = -\nabla p + \eta \nabla^2 \mathbf{v} + \mathbf{c}\phi, \tag{1}$$

$$\nabla \cdot \mathbf{v} = 0, \tag{2}$$

where ρ is fluid density, t time, $\mathbf{v}$ velocity, p pressure, η viscosity, and ϕ is the concentration field of HMPP in the fluid (liquid crystal). $\mathbf{c}\phi$ is the concentration-dependent force that generates fluid flows. Such force dependence is one of the simplest formulations that generates vortex-like fluid flow, as for example due to convection. We show in Supplementary Eq. 15 and in the Main text that similar formulation is possible also due to surface tension in wedged cells and possibly also due to mixing of the HMMP in the liquid crystal. The force dependence of $\mathbf{c}\phi$ is a minimal numerical model that recovers the experimental features, although multiple physical mechanisms can generate such flows, as discussed above. Additionally, we have checked that adding additional nematic stress tensor terms in the Navier Stokes equation does not result in a qualitative difference in the numerical results.

Convection-diffusion equation

The HMPP concentration field obeys the convection-diffusion equation

$$\frac{\partial \phi}{\partial t} + (\mathbf{v} \cdot \nabla)\phi = D \nabla^2 \phi, \tag{3}$$

where D is the diffusion constant for HMPP molecules in the liquid crystal. The units and the absolute magnitude of the concentration field have no direct role in Supplementary Eq. 3 due to its linearity. For simplicity, in numerical simulations, the concentration field is set to span between -1 and 1. Convective flows can be characterized by the Rayleigh number (Ra), which describes the ratio between the time scale for diffusive transport and the time scale for convective transport

$$\mathrm{Ra} = \frac{vl}{D}, \tag{4}$$

Here v is a characteristic velocity and l is the characteristic scale of the problem. In our case l is the height of the simulation area as defined in Supplementary Fig. 3.

Equations describing the realignment of the nematic liquid crystal due to flow

Nematic alignment is described by the unit orientation vector $\mathbf{n}$ and the degree of order S. The orientational dynamics equals

$$\frac{\partial \mathbf{n}}{\partial t} + (\mathbf{v} \cdot \nabla)\mathbf{n} - \frac{1}{2}(\nabla \times \mathbf{v}) \times \mathbf{n} = \frac{K}{\gamma} \nabla^2 \mathbf{n} + \lambda \mathbf{A}\mathbf{n} - \Lambda \mathbf{n}, \tag{5}$$

where the left hand side represents the corotational derivative of the orientational field $\mathbf{n}$, K is the elastic constant, γ the rotational viscosity, λ the parameter that sets the alignment of the orientational

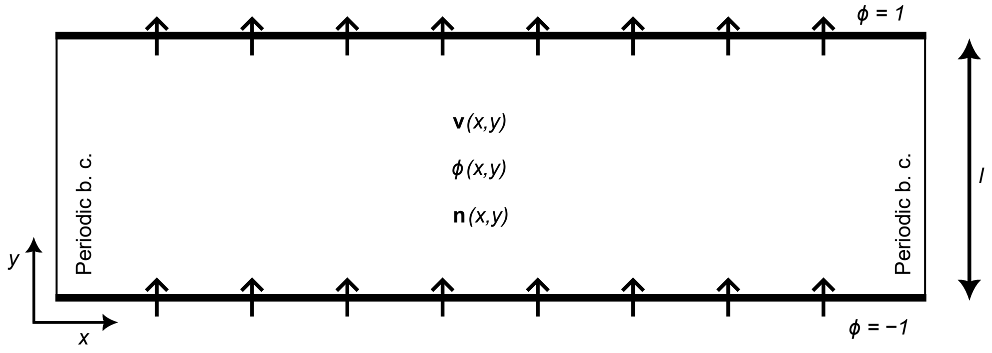

Supplementary Figure 3: Set-up of the numerical simulation. Periodic boundary conditions are set along the x-direction. An orientational field along the y-direction and $S = 1$ are set at $y = 0$ and $y = l$ along with the no-slip flow boundary conditions, and the concentration field of $\phi(y = 0) = -1$ and $\phi(y = l) = 1$. Velocity field $\mathbf{v}(x, y)$, concentration field $\phi(x, y)$, alignment field $\mathbf{n}(x, y)$, and the degree of order $S(x, y)$ are solved in time by a hybrid lattice Boltzmann numerical method.

field along the flow (for $\lambda > 1$) or tumbling motion of the orientational field (for $\lambda < 1$), $A_{ij} = (\partial_i v_j + \partial_j v_i)/2$ the symmetric velocity gradient tensor, and Λ the Lagrange multiplier constraining $|\mathbf{n}| = 1$. The dynamics of the degree of order equals

$$\frac{\partial S}{\partial t} + (\mathbf{v} \cdot \nabla)S = \frac{1}{\gamma}\left[K\nabla^2 S - KS(\nabla \mathbf{n})^2 - 4AS(S^2 - S_{\mathrm{eq}}^2)\right], \tag{6}$$

where A is a phase parameter tuning the potential that sets the degree of order towards the equilibrium value of S_{eq}. The dynamics of the orientational field (Supplementary Eq. 5) and the degree of order (Supplementary Eq. 6) is driven by the coupling with the flow field and by the relaxation towards a minimum of the free energy with the density

$$f = AS^2\left(S^2 - 2S_{\mathrm{eq}}^2\right) + \frac{KS^2}{2}(\nabla \mathbf{n})^2 + \frac{K}{2}(\nabla S)^2. \tag{7}$$

The above equations can be mapped to the tensorial descriptions of nematodynamics (such as Beris-Edwards model), but importantly preserve the vectorial symmetry that allows for the formation, stability, and dynamics of integer winding number two-dimensional defects.

Coupling of the alignment field with the velocity field is characterized by the Ericksen number (Er)

$$\mathrm{Er} = \frac{\gamma v l}{K}. \tag{8}$$

Simulation parameters

Simulations are performed on a $500\Delta x \times 100\Delta x$ numerical mesh, where Δx is the mesh resolution. Periodic boundary conditions are set in the x-direction. A no-slip boundary condition is set for the velocity field at $y = 0$ and at $y = l$, where $l = 100\Delta x$. The concentration field is set to $\phi = -1$ at $y = 0$ and $\phi = 1$ at $y = l$. An orientational field along the y direction is set at $y = 0$ and $y = l$. The simulation is performed with the time step of $\Delta t = 0.02\,(\Delta x)^2/D$. The following values of the nematic parameters are chosen throughout: $\lambda = 0.9$, $\eta = \gamma/2$, $S_{\mathrm{eq}} = 1$, $C = 0.1\,K/(\Delta x)^2$. Force term $\mathbf{c}$ points along y direction and its magnitude is chosen for each simulation to generate the appropriate Rayleigh and Ericksen numbers. Reynolds number $\mathrm{Re} = \frac{\rho v l}{\eta}$ is set to $\mathrm{Re} = 0.04\,\mathrm{Ra}$ in each simulation. Rayleigh and Ericksen numbers are specifically chosen for each simulation. The model equations are solved numerically by a hybrid lattice Boltzmann method, where specifically the Navier Stokes equations is solved using a lattice Boltzmann approach and the concentration and orientation dynamics is solved by a finite difference approach.

Surface tension forces in wedged geometry

In the studied experimental system, vortex flows are an important structural feature that drives the deformation of the nematic orientational field. In numerical simulations, vortex flows are generated by an appropriate force density in the Navier-Stokes equation. Here, we discuss how such force density could arise due to surface tension. Surface tension is pivotal in Bernard-Marangoni convection cells, which are generated due to temperature gradiets and temperature-dependent surface tension. Here, we consider the photoinitiator used in experiments as a surface active agent that alters the surface tension and derive the force density due to the varying concentration of the photoinitiator and the variation of the cell thickness in a wedged geometry (Supplementary Fig. 4).

To derive the forces acting on a volume of fluid in a wedged cell, we consider a volume element $\mathrm{d}V$ of height h with the surface tension acting upon the surface element $\mathrm{d}S = \mathrm{d}V/h$ with the free energy of $\mathrm{d}F = \sigma \mathrm{d}S$, as shown by Supplementary Fig. 4. We consider thin cells, for which there is no variation of the concentration field along z. We write the change of the free energy when the volume element is displaced by $\delta\mathbf{r} = (\delta x, \delta y)$:

$$\delta \mathrm{d}F = -\nabla\sigma \cdot \delta\mathbf{r}\, \mathrm{d}S + \sigma\, \delta \mathrm{d}S \tag{9}$$

$$= -\frac{\partial\sigma}{\partial\phi}\mathrm{d}S\, \nabla\phi \cdot \delta\mathbf{r} - \sigma\frac{\mathrm{d}V}{h^2}\nabla h \cdot \delta\mathbf{r}, \tag{10}$$

$$\delta\frac{\mathrm{d}F}{\mathrm{d}S} = -\left[\frac{\partial\sigma}{\partial\phi}\nabla\phi + \frac{\sigma}{h}\nabla h\right] \cdot \delta\mathbf{r} \tag{11}$$

$$= -\mathbf{f} \cdot \delta\mathbf{r}, \tag{12}$$

where the first term considers the Marangoni force of the neighbouring fluid due to concentration-dependent surface tension and the second term acknowledges the fact that if the volume element is moved by $\delta\mathbf{r}$, its surface element changes due to incompressibility for $\delta \mathrm{d}S = -\frac{\mathrm{d}V}{h^2}\delta h = -\frac{\mathrm{d}S}{h}\nabla h \cdot \delta\mathbf{r}$.

The surface force density in Supplementary Eq. 12 therefore includes both effects of concentration gradients and height gradients in a wedged cell:

$$\mathbf{f} = \frac{\partial\sigma}{\partial\phi}\nabla\phi + \frac{\sigma}{h}\nabla h. \tag{13}$$

For simplicity, we assume a linear surface equation of state [1]

$$\sigma = \sigma_0 + k\phi, \tag{14}$$

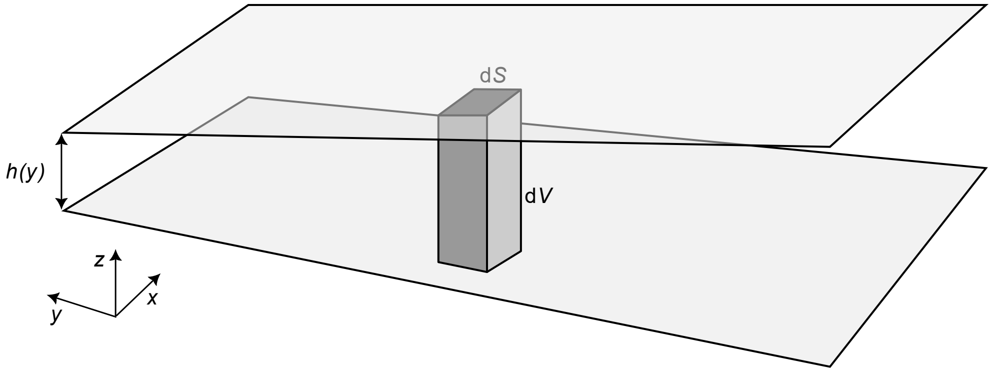

Supplementary Figure 4: Fluid inside a wedged cell. A surface active agent with a concentration field $\phi(x, y)$ is dispersed inside a cell. We consider the variation of the free energy as a volume of fluid is displaced in the xy plane.

where σ_0 is the surface tension with no active agent present, $k < 0$ corresponds to surfactants that decrease the surface tension and $k > 0$ corresponds to surface active agents that increase the surface tension. Using Supplementary Eq. 14, we can rewrite the force density as

$$\mathbf{f} = k\nabla\phi + \frac{\sigma_0}{h}\nabla h + \frac{k}{h}\phi\nabla h. \tag{15}$$

Supplementary Equation 15 describes the surface force density due variations of thickness and concentration of the surface active agent in a wedged geometry. The last term in Supplementary Eq. 15 is directed along ∇h and is proportional to the concentration. Its sign is important when considering if such a force field could lead to a instability and formation of vortex flows. Supplementary Fig. 5 shows that for $k > 0$, a local fluctuation in the concentration field can in principle start a formation of a vortex flow. The regime of $k > 0$ corresponds to surface active agents that increase the surface tension with increased concentration. Surface tension measurements in experiments (see Supplementary Note 4) reveal that HMPP acts as a surfactant with $k < 0$ and a mechanism as shown in Supplementary Fig. 5 is most likely not directly responsible for formation of vortex flow.

The calculation of the surface force can be used to compare them to the gravity effects if we consider how an element of fluid lowers its center of mass as it is moved in a wedged cell. For a wedged cell with a flat upper interface (Supplementary Fig. 4), the gravity contribution to Supplementary Eq. 13 equals

$$\mathbf{f}_{\mathrm{g}} = \frac{\rho(\phi) g h \nabla h}{2}, \tag{16}$$

where g is the gravitational acceleration. For typical value of $h \sim 5\,\mu\mathrm{m}$ and concentration-dependant density and surface tension changes $\Delta\rho \sim 15\,\mathrm{kg\,m^{-3}}$ and $\Delta\sigma \sim 0.0015\,\mathrm{N\,m^{-3}}$, we can compare the magnitudes of Supplementary Eq. 13 and Supplementary Eq. 16 and observe that the force density due to gravity is multiple orders of magnitude smaller than the force density due to the surface tension.

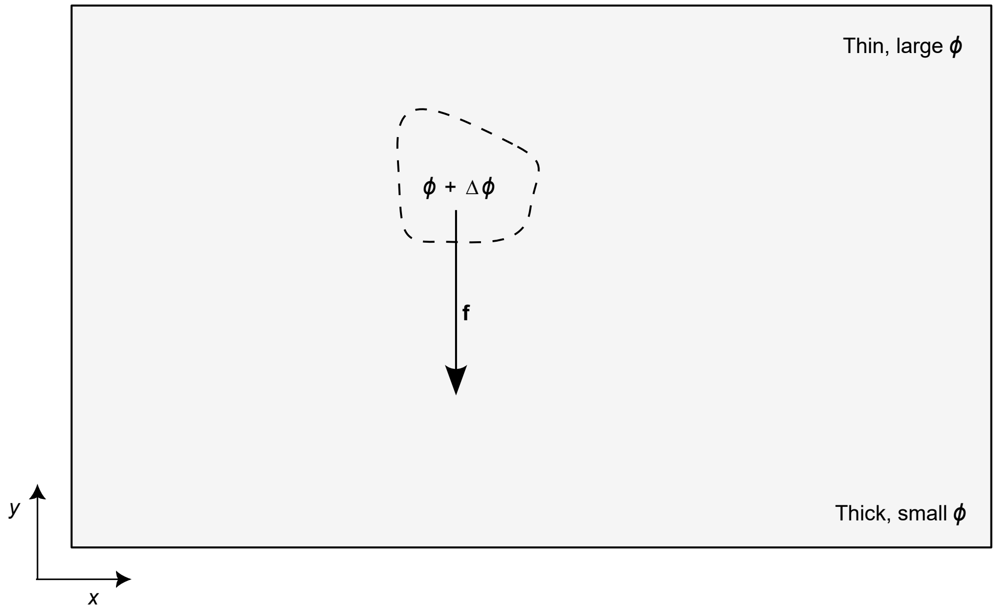

Supplementary Figure 5: Top view of a wedged cell with a large concentration field at the thin part of the cell and a smaller concentration at the thick part. For surface active agents that increase the surface tension ($k > 0$), a local fluctuation of an increased concentration will have a larger force applied compared to the surrounding fluid and will be convected along the $-y$ direction. This mechanism could lead to an instability and formation of vortex patterns. Large concentration at the thin part of the cell occurs due to the material diffusing from the bottom layer into the wedged cell.

SUPPLEMENTARY NOTE 4:
Surface tension measurements

In order to better understand the physics behind the continuous generation of flow and crossover to turbulence observed in our experiments, one needs to identify the forces which are driving the fluid into the flow. One of the possible driving forces is the Marangoni force that emerges due to the gradient of the interfacial tension and transfers the mass along the interface. In our case the gradient of surface tension could be a result of the gradient of HMPP concentration. We therefore measured the surface tension of water and 8CB liquid crystal (LC) containing different concentrations of the HMPP photoinitiator as well as the interfacial tension at the interface between the 8CB and water.

Our method is based on Laplace pressure difference due to surface tension that emerges between the outside and the inside of the curved interface between two bordering materials. The Laplace pressure is equal to

$$\Delta p = \gamma(\frac{1}{R_1} + \frac{1}{R_2}), \tag{17}$$

where γ is the surface (or interfacial) tension and R_1, R_2 are the radii of two principal curvatures.

The measurements of Laplace pressure difference were made in a round glass capillary, tapered to an outer diameter of 245 µm. The tip of the capillary was filled with water or 8CB through capillary suction. The capillary was attached to the microfluidic flow controller (Elveflow, OB1, 0-200 mbar), with which pressures of a few millibar could be applied with a 0.03 mbar precision.

The part of the capillary that was not filled with either water or 8CB, was filled with air, which filled the tubing to the pressure generation of the flow controller. The capillary was positioned horizontally (or in some cases at a small angle - up to 5°), above the microscope objective, to observe the shape of the

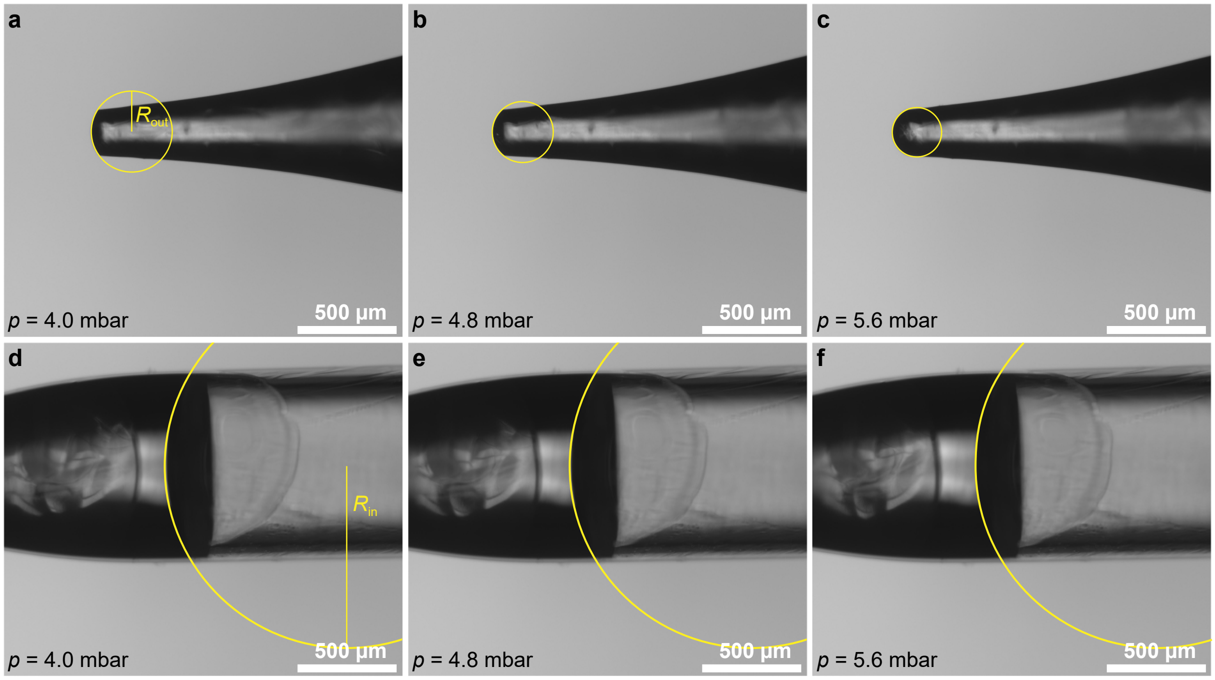

Supplementary Figure 6: Measurement of the radii of curvature at different applied pressures. (a–c) At the curved surface at the capillary opening we measure R_{out}. (d–f) Corresponding measurements of R_{in} from the curved surface, formed inside the capillary, where the liquid is in contact with pressurized air from the flow controller.

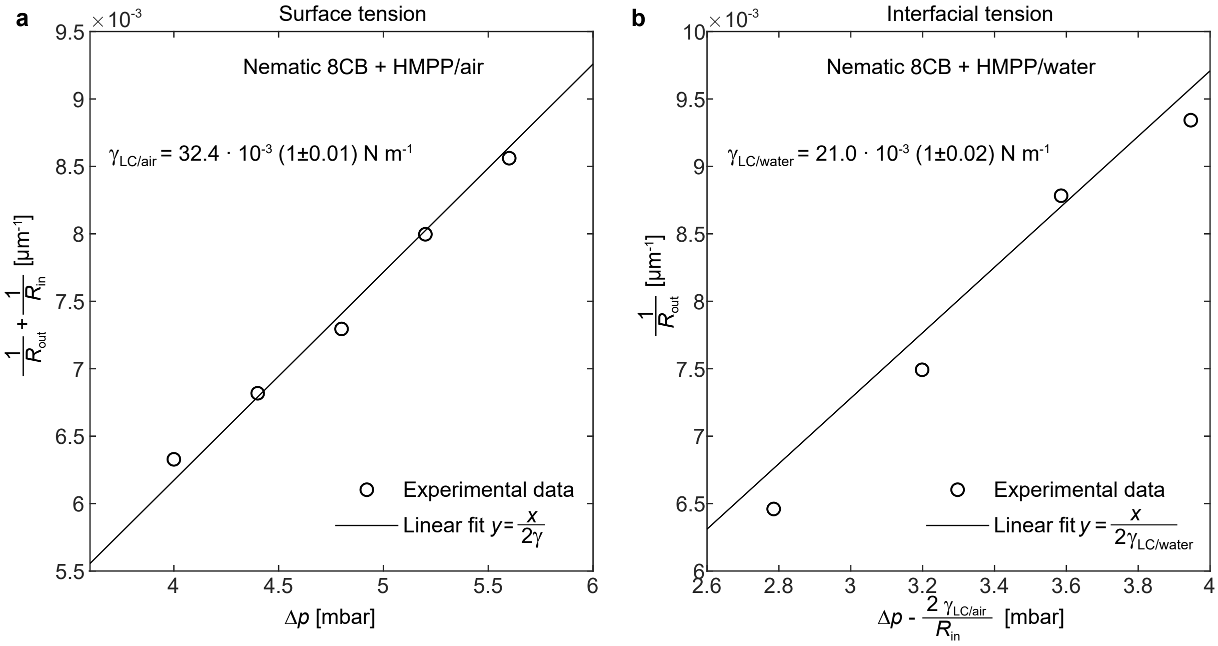

Supplementary Figure 7: Typical measurements of a surface tension using the method of Laplace pressure difference. (a) Measurement of the sum of the curvatures of both interfaces for the nematic phase of 8CB with 3.5% HMPP (2-hydroxy-2-methylpropiophenone photoinitiator) at room temperature and in contact with air. The slope gives the surface tension of 8CB/HMPP solution in contact with air. (b) When we measure the interfacial tension of the LC in contact with water, the liquid crystal (LC) has two different curved surfaces in contact with two different materials. To obtain the interfacial tension $\gamma_{\mathrm{LC/water}}$ from a linear fit, $\gamma_{\mathrm{LC/air}}$ has to be known beforehand for the same LC, i.e. it is taken from data in (a).

curved surface of liquid that was inflated by applying an additional pressure. As the capillary was only partially filled, two surfaces were formed: one at the tip, where the material was in contact with an outer medium (air, water or water with HMPP) and another one inside the capillary, where the material was in contact with pressurized air. Pressure in the capillary was increased in steps of 0.4 mbar when measuring the surface (or interfacial) tension of LCs and 1.0 mbar for the measurements of surface tension of water. We made sure that at each pressure value the liquid was stable. In each pressure step, we measured radii of curvature for both curved surfaces, as shown in Supplementary Fig. 6. Due to the cylindrical symmetry of the capillary the two principal curvatures of a surface are taken equal, and the shape of two liquid interfaces is considered spherical at both ends.

In each set of measurements we obtained the surface (interfacial) tension from a linear fit to data (see Supplementary Fig. 7). When measuring the surface tension, both curved surfaces are in contact with air. In this case

$$\Delta p = \Delta p_{\mathrm{out}} + \Delta p_{\mathrm{in}} = \frac{2\gamma}{R_{\mathrm{out}}} + \frac{2\gamma}{R_{\mathrm{in}}} = 2\gamma(\frac{1}{R_{\mathrm{out}}} + \frac{1}{R_{\mathrm{in}}}). \tag{18}$$

Therefore, γ is obtained by fitting a line to a sum of inverse radii $1/R_{\mathrm{out}} + 1/R_{\mathrm{in}}$ vs. the pressure difference Δp, as shown in Supplementary Fig. 7(a).

When we want to measure the interfacial tension of LC in contact with water, we have the outer (inflated) curved surface in contact with water and the inner one in contact with air:

$$\Delta p = \Delta p_{\mathrm{out}} + \Delta p_{\mathrm{in}} = \frac{2\gamma_{\mathrm{LC/water}}}{R_{\mathrm{out}}} + \frac{2\gamma_{\mathrm{LC/air}}}{R_{\mathrm{in}}}. \tag{19}$$

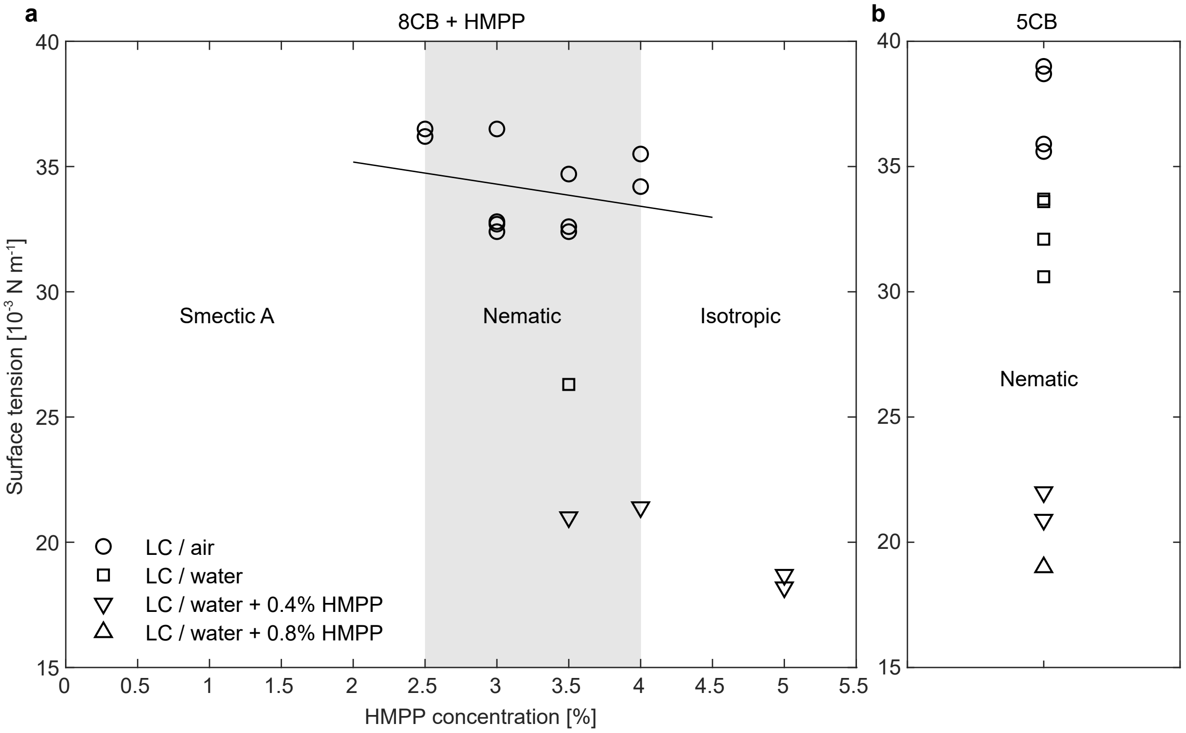

Supplementary Figure 8: Surface tension of 8CB with HMPP (2-hydroxy-2-methylpropiophenone photoinitiator) and of 5CB. (a) Surface tension of 8CB with different concentrations of HMPP. For 3.5% the plot also shows interfacial tension of 8CB/HMPP in contact with water and water/0.4% HMPP. The shaded region indicates the nematic phase. (b) Surface tension of 5CB when in contact with air, water, water/0.4% HMPP, water/0.8% HMPP. LC - liquid crystal.

Therefore, $\gamma_{\mathrm{LC/air}}$ has to be known beforehand to calculate the interfacial tension $\gamma_{\mathrm{LC/water}}$ from a linear fit as shown in Supplementary Fig. 7(b).

In Supplementary Fig. 8(a) we present the results of the surface tension measurements in the nematic and isotropic phases of 8CB which are obtained for various concentrations of added HMPP. When the concentration of added HMPP in 8CB/HMPP solution is in the range from 2.5% to 4%, the 8CB is in the nematic phase at room temperature, where the experiments were performed. If the concentration of added HMPP is increased above 5%, the 8CB is in the isotropic phase at room temperature. For comparison we also performed measurements with 5CB - a room temperature nematic - in contact with air, water and water with the addition of HMPP, which is shown in Supplementary Fig. 8(b).

We see from Supplementary Fig. 8(a) (open circles) that surface tension of 8CB/HMPP in the nematic phase in contact with air only slightly decreases with increasing concentration of the HMPP. It is in the range around $35\,\mathrm{mN\,m^{-1}}$, which is a typical surface tension for nematic-air interface. Although the measurements in Supplementary Fig. 8(a, b)appear quite scattered, the method of measuring Laplace pressure difference when inflating a liquid droplet proves quite usefull. It gives very indicative information on the trend of surface tension of LCs when surfactants are added.

The measured interfacial tension of 8CB/HMPP (3.5% HMPP) in contact with water (one open square at $\sim$3.5%) is much lower than that, i.e. $26\,\mathrm{mN\,m^{-1}}$, and gets even lower when 0.4% HMPP is added to water. Here, we need to mention that while the starting concentration of HMPP in the LC was 3.5%, it changed on the time scale of seconds after submerging the capillary under pure water. The open triangles in Supplementary Fig. 8(a) represent the measurements of surface tension of 8CB/HMPP in contact with water containing 0.4% of HMPP. One can see from these data that by adding HMPP

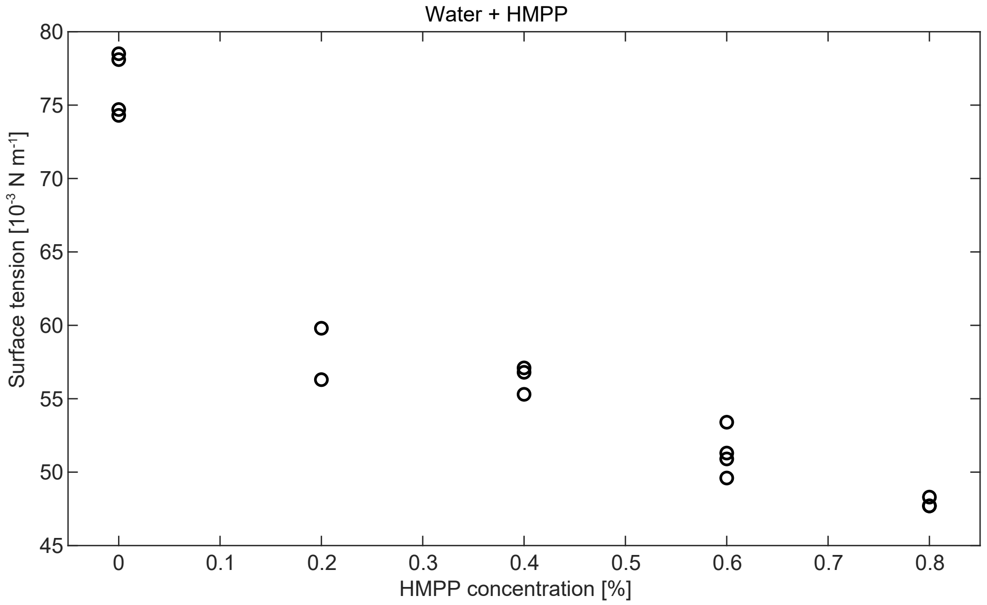

Supplementary Figure 9: Surface tension of water in dependence of the HMPP (2-hydroxy-2-methylpropiophenone photoinitiator) concentration.

to the LC and water significantly lowers the LC-water interfacial tension, which drops to $\sim 20\,\mathrm{mN\,m^{-1}}$. We noticed during these measurements that if the water already contained some HMPP, the LC near the junction would undergo a phase transition into an isotropic phase due to diffusion of HMPP from water to the LC. On the other hand, if the water was free of HMPP, the LC would change its phase into smectic-A in a few tens of seconds. This indicates that HMPP partially difused from LC to water. The lowering of the LC-water interfacial tension by adding HMPP is also seen for 5CB in Supplementary Fig. 8(b). Here, the surface tension of pure 5CB in contact with air is slightly larger than in 8CB/HMPP, i.e. around $37\,\mathrm{mN\,m^{-1}}$. The interfacial tension at the 5CB/water contact drops to $32\,\mathrm{mN\,m^{-1}}$, and even lower, when HMPP is added to water.

Further, in Supplementary Fig. 9 we show how an addition of HMPP lowers surface tension of water. At 0.4% HMPP concentration that was used in the experiments with topological defect formation, the surface tension of water is cca. $57\,\mathrm{mN\,m^{-1}}$.

Taking all these surface tension measurements into account, we present in Supplementary Fig. 10 a schematic drawing of the concentration distribution in the experimental sample (the thin wedge-like film floating on water), along with the surface tension values. At the very contact between water and LC, the LC is in the isotropic phase, as the HMPP concentration close to the line is larger than 4%. The concentration of HMPP in 8CB decreases as we go further from the water/LC line until we reach the isotropic-nematic line, where the HMPP concentration reaches 4.0%. By further going along the 8CB wedge, the concentration of HMPP lowers until we reach the 2.5% of HMPP in 8CB, at which point the 8CB becomes smectic. It is important to note that the surface tension of 8CB/HMPP with respect to air increases as we go from the isotropic-nematic line to the nematic-smectic line, i.e. along increasing thickness of the nematic wedge. This will be important for the analysis of concentration-induced flow of 8CB/HMPP along the air interface.

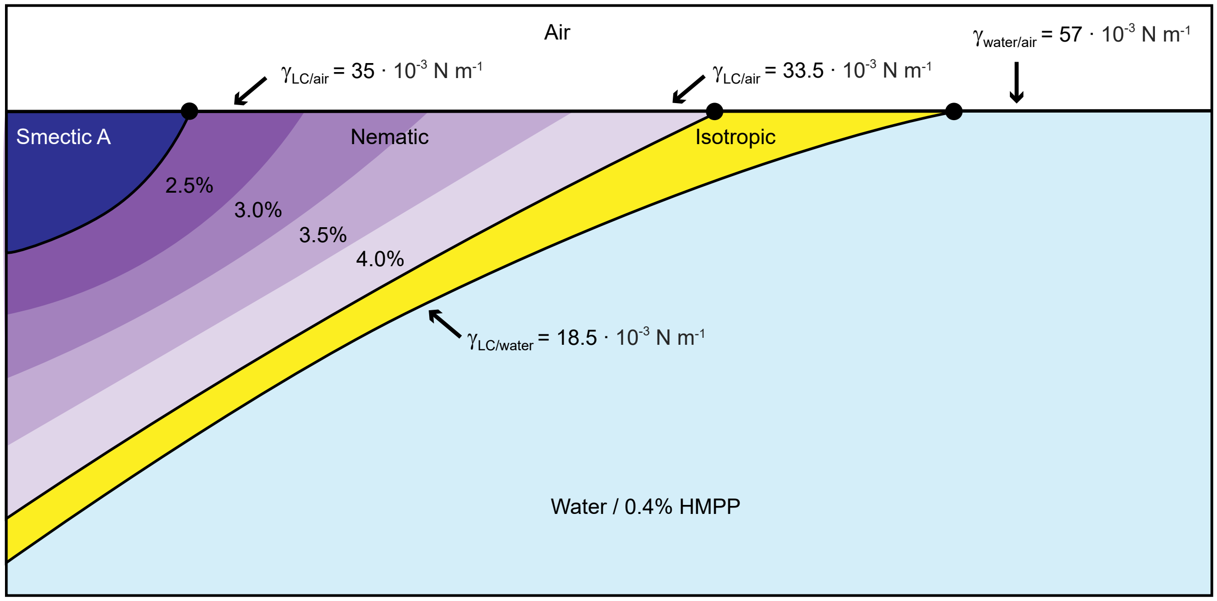

Supplementary Figure 10: Schematic drawing of the concentration distribution within the thinning film. Note that the surface tension increases, as we move from thin to thicker part of nematic 8CB/HMPP wedge. HMPP – 2-hydroxy-2-methylpropiophenone photoinitiator, LC – liquid crystal.

Supplementary References

[1] Pozrikidis, C. *Introduction to Theoretical and Computational Fluid Dynamics* (Oxford University Press, New York, 2011).
