# Supplementary material for: Continuous generation of topological defects in a passively driven nematic liquid crystal
Source: Nat Commun. 2022 Nov 11;13:6855. doi: 10.1038/s41467-022-34384-5 (PMC9652398; doi:10.1038/s41467-022-34384-5)
Supplement: Supplementary file 3 — Description of Additional Supplementary Files [file 41467_2022_34384_MOESM3_ESM.pdf]

Description of Additional Supplementary Files

Supplementary Movie 1
A drop of smectic A liquid crystal is submerged in water containing 0.4 wt% of 2-hydroxy-2-methylpropiophenone photoinitiator (HMPP). The first part of the movie shows a fast-forward (10x speed) recording of the drop slowly reshaping to minimize its interface with water. At the same time the HMPP is diffusing into the droplet, causing the LC to undergo a phase transition from smectic A to nematic phase. This can be seen as a change in the LC texture from the highly scattering smectic A texture to the smoother nematic texture (the black edge is an optical artifact due to lensing of the drop). At approximately 9 seconds into the movie, it is slowed down to normal speed, showing the ‘explosion’ when a wide band of the turbulent nematic appears around the drop. Defects can be seen appearing as dark spots.

Supplementary Movie 2
Defects are spontaneously forming and annihilating in the thin nematic film spreading outward from the 8CB drop along the surface of the aqueous environment. In brightfield microscopy they can be seen as dark spots, pairs of which are connected with lines. They can be seen appearing and later annihilating spontaneously. When crossed polarizers are introduced, the defects are easier to observe, each as a set of four dark brushes emerging from a single point. The colorful concentric fringes in the nematic band tell us the film is changing in thickness, thinning as it spreads further away from the core LC drop. In the end we show also a red-plate imaging of the phenomenon. Director orientation in the nematic film can be discerned from it as shown in the schematics.

Supplementary Movie 3
This movie shows four rare events of defect formation in the middle of the nematic film recorded under crossed polarizers. In each event two defects are created at the same time, obeying the total topological charge conservation. The speed is reduced to 0.2x of the original speed.

Supplementary Movie 4
We show time development of the nematic film in a red-plate imaging technique. A thin hybrid nematic layer is passively driven by the diffusion of HMPP molecules. Gradually it takes on a periodic domain configuration. Blue and yellow regions correspond to domains of oppositely tilted LC molecules, forming a herring-bone pattern. Later, inside the primary domains islands of opposite orientations appear. As the LC film thickens with time, the director field becomes more chaotic and changes ever more rapidly. The broader range of colors is due to the thicker nematic film introducing higher orders of retardation. At last, when the director field dynamics grows even more complex, defects start forming and annihilating. Sections of the movie are fast-forwarded at 20x speed. The speed is reduced to normal at the occasions corresponding to Figure 3(a-d).

Supplementary Movie 5
Three typical defect creation events at the outer edge of the nematic film are recorded in the red-plate imaging technique. Event 1 corresponds to the event represented and thoroughly explained in Figure 4(a-l). The speed is reduced to 0.2x of the original speed.

Supplementary Movie 6
Formation of stable vortex flows and periodic nematic distortion. The simulation starts from a random concentration field, zero flow, and uniform alignment. The video duration equals $2000\ (\Delta x)^2/D$. The numerical simulation is performed at Ra ≈ 60 and Er ≈ 12. The top panel

shows the concentration field (colormap) and the flow field (black arrows). The middle panel shows the degree of order (colormap) and the alignment field (black arrows). The bottom panel shows the alignment angle in colormap that compares well to the lambda-plate polarization microscopy. A snapshot of this movie is presented in Fig. 6.

Supplementary Movie 7

Nematic deformation at higher flow magnitudes for Ra ≈ 300 and Er ≈ 60. The initial condition is the final stable configuration from Supplementary Video 6. The video duration equals $2000\ (\Delta x)^2/D$. The flow is strong enough to generate larger distortions of the nematic alignment, but does not produce any topological defects.

Supplementary Movie 8

Irregular flow profiles and defect dynamics. The initial condition is the final stable configuration from Supplementary Movie 6. The video duration equals $1000\ (\Delta x)^2/D$. The numerical simulation is performed at Ra ≈ 650 and Er ≈ 130. Nematic defects show many creation and annihilation events with a complex dynamics of the flow-induced nematic distortions. A snapshot of this movie is presented in Fig. 7.
